# Müllerian Agenesis Presenting as Primary Amenorrhea in a 16‐Year‐Old Girl From a Low‐Resource Setting in Bangladesh: Psychological Impact and Multidisciplinary Management

**DOI:** 10.1002/ccr3.73132

**Published:** 2026-07-10

**Authors:** Iftekhar Ahmed Sakib, Musarrat Sultana Shumi, Rozina Sultana, Ayesa Perveen, Nishad Tasnim, Shirazum Munira

**Affiliations:** ^1^ Shaheed Suhrawardy Medical College and Hospital Dhaka Bangladesh; ^2^ Dhaka Medical College and Hospital Dhaka Bangladesh; ^3^ OBGYN, Shaheed Suhrawardy Medical College and Hospital Dhaka Bangladesh; ^4^ Manikganj Medical College and Hospital Manikganj Dhaka Bangladesh

**Keywords:** adolescence, counseling, karyotype, reconstruction, Vaginoplasty

## Abstract

Müllerian agenesis (MRKH syndrome) causes primary amenorrhea in phenotypically normal females. We report a 16‐year‐old girl with normal secondary sexual characteristics, a short blind vagina, absent uterus on ultrasonography, and a 46,XX karyotype. Successful vaginoplasty and counseling in a low‐resource setting highlight the importance of systematic evaluation and multidisciplinary management.

## Key Clinical Message

Müllerian agenesis should be suspected in adolescents presenting with primary amenorrhea despite normal development of secondary sexual characteristics. Careful clinical examination, ultrasonography, and karyotyping allow accurate diagnosis even in low‐resource settings. Multidisciplinary management—including vaginoplasty, psychological counseling, and family engagement—is essential to achieve satisfactory anatomical, functional, and psychosocial outcomes.

**Abbreviations**
AIS: Androgen Insensitivity Syndrome
IVF: In Vitro Fertilization
MRI: Magnetic Resonance Imaging
MRKH: Mayer–Rokitansky–Küster–Hauser syndrome

## 1 Introduction

Müllerian agenesis, commonly referred to as Mayer–Rokitansky–Küster–Hauser (MRKH) syndrome, is a rare congenital anomaly affecting the female reproductive tract. It occurs in approximately 1 in 4,500–5,000 female births and represents one of the most common causes of primary amenorrhea in adolescents with otherwise normal secondary sexual characteristics [1, 2]. The condition results from failure of embryological development of the Müllerian ducts during early fetal life, leading to congenital absence or severe hypoplasia of the uterus and the upper portion of the vagina.

Despite the absence of the uterus, individuals with MRKH syndrome typically have normally functioning ovaries, normal female external genitalia, and a normal female karyotype (46,XX). Because ovarian endocrine function is preserved, secondary sexual characteristics such as breast development and pubic hair distribution develop normally during puberty. As a result, affected individuals often remain undiagnosed until adolescence, when they seek medical attention for primary amenorrhea [3].

MRKH syndrome is generally classified into two major types. Type I, also referred to as the typical form, involves isolated uterovaginal agenesis without associated systemic anomalies. Type II, sometimes called atypical MRKH or MURCS association (Müllerian duct aplasia, renal dysplasia, and cervicothoracic somite anomalies), includes associated renal, skeletal, or other congenital abnormalities [2].

The primary goals of management in MRKH syndrome include the creation of a functional vagina to allow satisfactory sexual activity and preservation of psychological well‐being. Treatment options range from non‐surgical vaginal dilation methods to various surgical procedures designed to construct a neovagina. International guidelines generally recommend non‐surgical dilation as first‐line therapy in motivated patients; however, surgical vaginoplasty remains an effective alternative when conservative methods are not feasible or unsuccessful [4, 5].

In high‐resource healthcare systems, multidisciplinary management involving gynecologists, psychologists, reproductive specialists, and genetic counselors is recommended to address the medical, psychological, and reproductive aspects of the condition [6, 7]. However, in many low‐ and middle‐income countries, such comprehensive services may not be readily available due to limited healthcare infrastructure, diagnostic resources, and trained personnel [8].

Beyond its anatomical implications, MRKH syndrome carries substantial psychosocial consequences. Adolescents diagnosed with the condition frequently experience emotional distress related to infertility, concerns about sexual identity, and anxiety regarding future relationships and marriage [6, 9].

These psychosocial challenges may be particularly pronounced in conservative societies where menstruation and fertility are closely linked to perceptions of womanhood and marital eligibility. In countries such as Bangladesh, reproductive capability is often considered an essential component of female identity, and the inability to menstruate may lead to social stigma, family concern, and psychological distress [10].

## 2 Case Presentation

### 2.1 Patient Information

A 16‐year‐old unmarried female presented to the gynecology outpatient department with the chief complaint of **absence of menstruation since puberty**. She reported normal development of breasts and pubic hair beginning around the age of 12 years. There was no history of cyclic abdominal pain, urinary symptoms, or bowel disturbances.

The patient belonged to a lower‐middle‐income family living in a semi‐urban area of Bangladesh. There was no known family history of congenital reproductive anomalies or delayed puberty. Her parents sought medical evaluation because of increasing concern regarding the delayed onset of menstruation.

### 2.2 Clinical Findings

General physical examination revealed a healthy adolescent female with a normal female body habitus. Secondary sexual characteristics were well developed. Breast development corresponded to Tanner stage V, and pubic hair distribution was consistent with the normal female pattern.

External genital examination revealed normal female genitalia. On gentle vaginal examination, a short blind vaginal pouch measuring approximately **2.5 cm** in length was identified. No cervix or uterine structure could be palpated. Rectal examination suggested the absence of a well‐formed uterus.

### 2.3 Diagnostic Assessment

Diagnosis of Müllerian agenesis requires a systematic and comprehensive evaluation integrating clinical examination, imaging studies, and cytogenetic analysis [4].

**FIGURE 1:**
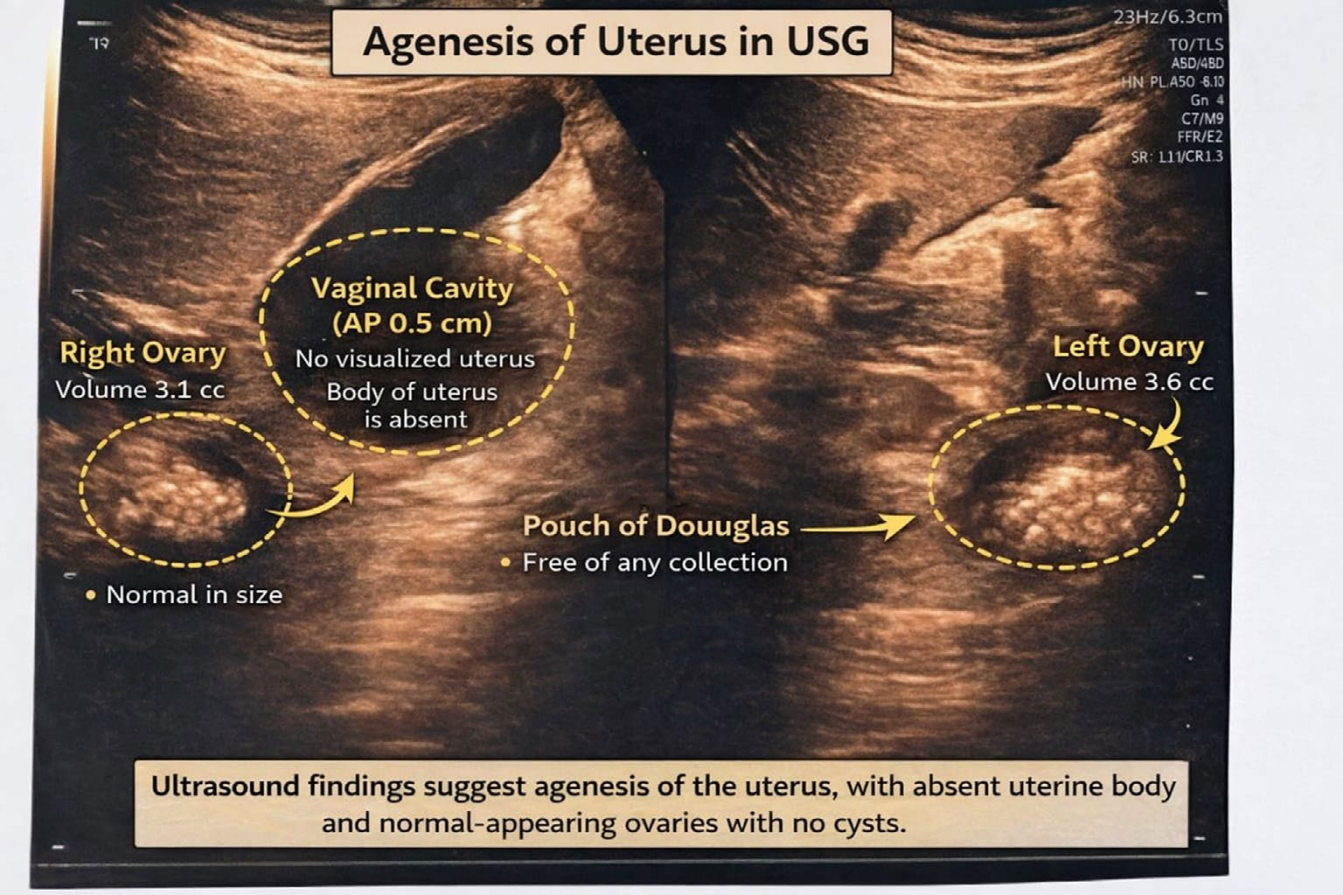
Pelvic ultrasonography of the patient. Transabdominal ultrasonography of the pelvis demonstrates key features of Müllerian agenesis in the present case. Uterus: Not visualized. Only a linear hypoechoic structure (anteroposterior diameter 0.5 cm) is seen, representing the blind vaginal pouch. Ovaries: Bilaterally normal in size and echotexture (Right ovary volume 3.1 cc, Left ovary volume 3.6 cc). Pouch of Douglas: Free of fluid collection. Additional findings: No appendicular or pelvic masses, ascites, pleural effusion, or lymphadenopathy detected. These findings demonstrate the absence of the uterine body with preserved ovarian morphology, correlating with clinical primary amenorrhea and supporting the diagnosis of complete Müllerian agenesis.

Pelvic ultrasonography was performed and demonstrated the **absence of the uterus**, with both ovaries appearing normal in size and morphology. These findings suggested uterine agenesis with preserved ovarian function [11] (Figure 1).

**FIGURE 2:**
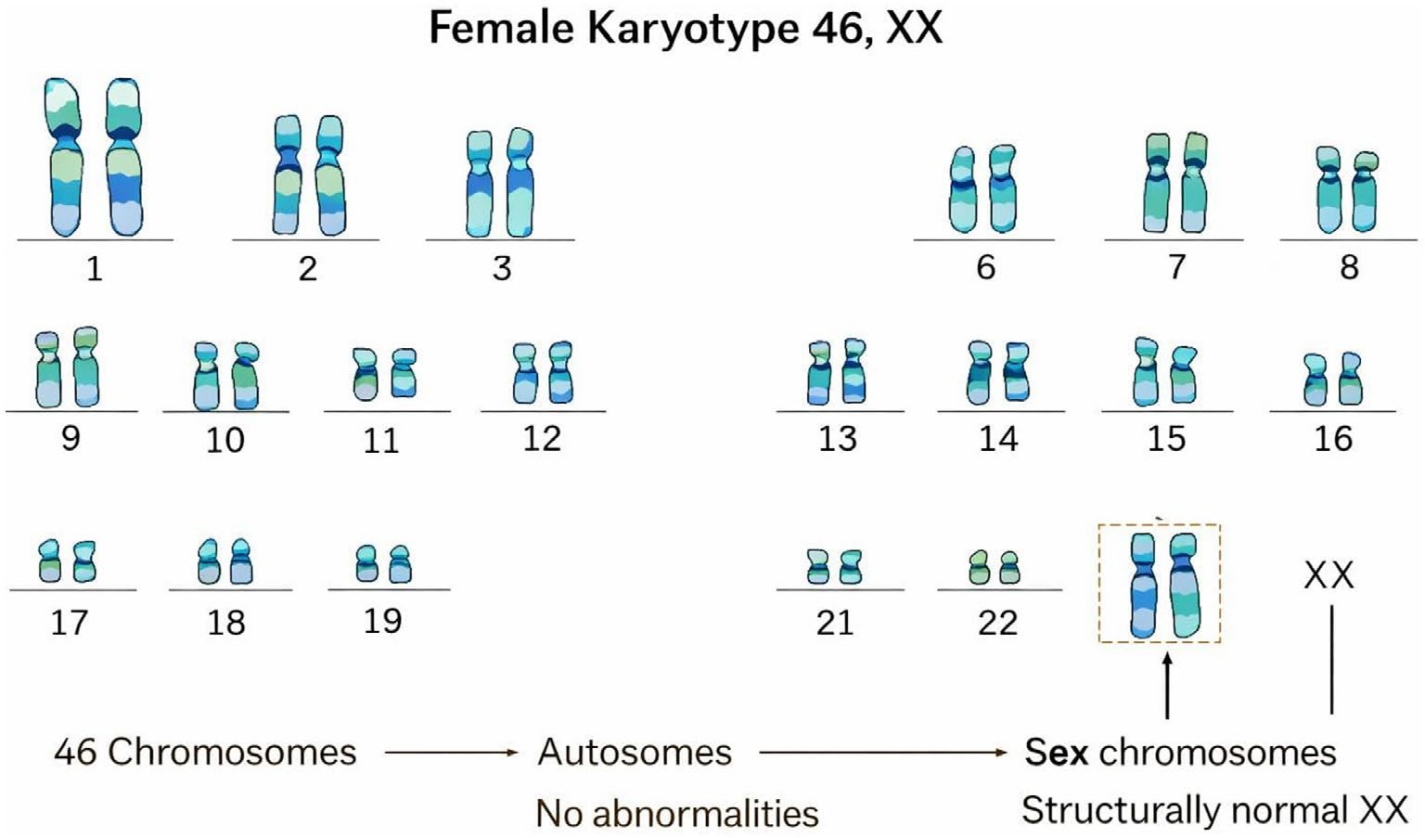
Conventional G‐banded karyotype showing a normal female chromosomal complement (46,XX). G‐banded metaphase karyotype prepared from peripheral blood lymphocytes demonstrating a normal female chromosomal complement. The total chromosome number is 46, with two structurally normal X chromosomes. All autosomes (chromosomes 1–22) are present in normal pairs without detectable numerical or structural abnormalities at the resolution of conventional cytogenetic analysis. Karyotype designation: 46,XX.

Cytogenetic analysis (karyotyping) revealed a **46,XX chromosomal complement**, confirming a normal female genetic pattern and excluding conditions such as complete androgen insensitivity syndrome [3, 12] (Figure 2).

Magnetic resonance imaging was not performed due to financial and resource constraints. However, the available clinical, ultrasonographic, and cytogenetic findings were sufficient to establish the diagnosis of **complete Müllerian agenesis (MRKH Type I)**.

### 2.4 Treatment

The patient and her family were counseled regarding the nature of the condition and available treatment options. After discussion of both non‐surgical and surgical approaches, surgical vaginoplasty was chosen due to the patient's preference and the availability of surgical expertise.

**FIGURE 3:**
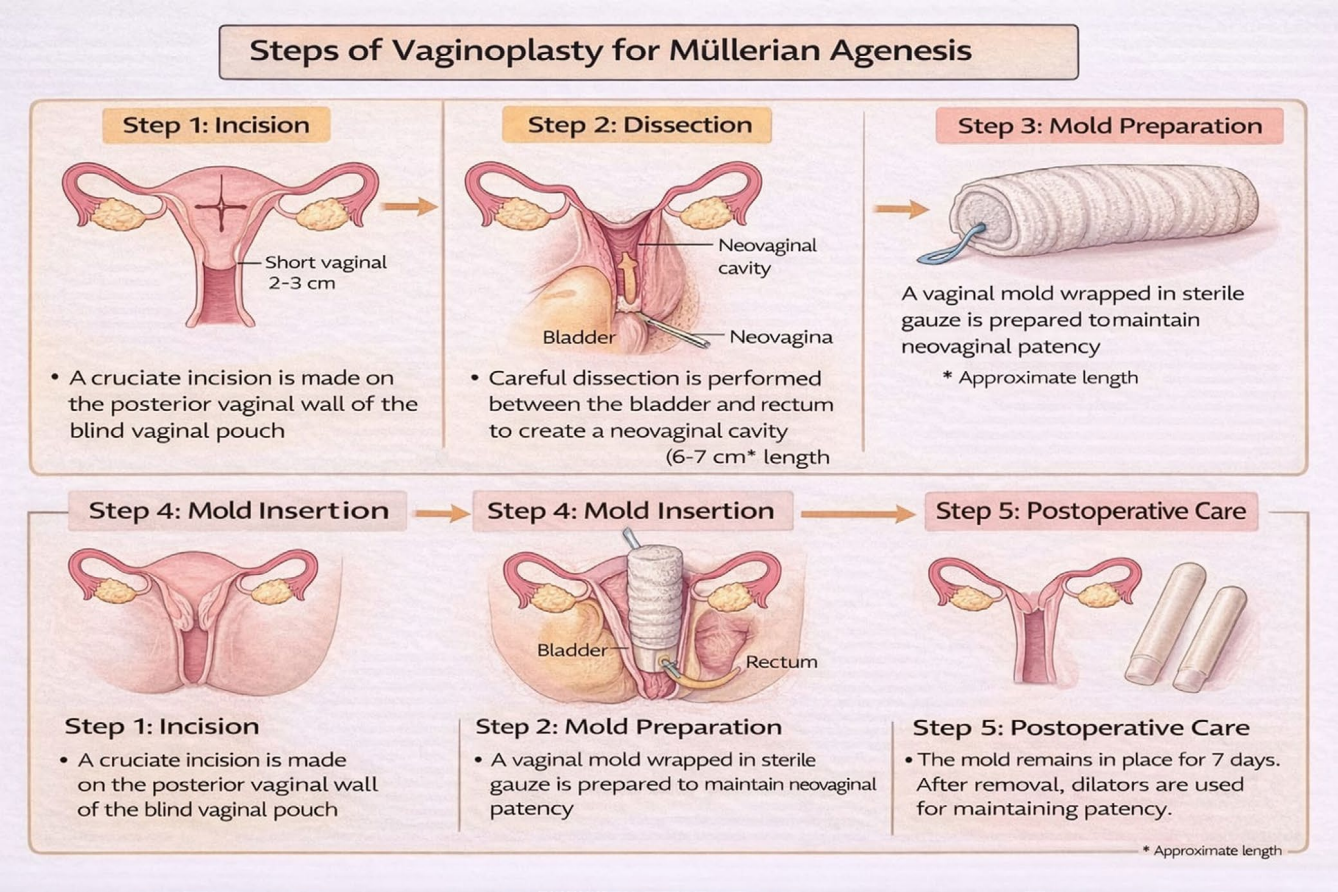
Schematic illustration of the steps of vaginoplasty in Müllerian agenesis. Stepwise depiction of vaginoplasty for patients with Müllerian agenesis: Step 1: Incision—Cruciate incision over the posterior vaginal wall of the blind vaginal pouch (2–3 cm). Step 2: Dissection—Creation of a neovaginal cavity by careful dissection between the bladder and rectum (6–7 cm). Step 3: Mold Preparation—Vaginal mold prepared using sterile gauze, foam, and a condom to maintain neovaginal patency. Step 4: Mold Insertion—Placement of the vaginal mold in the neovagina and apposition of the vagina; Foley catheter inserted for urinary drainage. Step 5: Postoperative Care—Mold retained for 7 days, followed by ongoing dilator therapy to maintain patency. Annotations highlight relevant anatomical structures, including the bladder, rectum, neovagina, and mold. This schematic provides a visual guide to surgical steps and postoperative management in resource‐limited settings.

The procedure involved the creation of a neovaginal canal through dissection between the bladder and rectum. A vaginal mold was constructed using sterile gauze and condom material to maintain patency of the newly created vaginal cavity. The mold was inserted intraoperatively and maintained postoperatively to support epithelialization of the neovaginal lining (Figure 3).

### 2.5 Follow‐Up and Outcomes

The postoperative period was uneventful. The patient was advised to continue regular use of vaginal dilators to maintain vaginal length and patency.

Follow‐up visits at one and three months demonstrated satisfactory healing of the neovaginal canal with no evidence of infection or stenosis. Psychological counseling sessions were continued to help the patient adapt to the diagnosis and understand future reproductive possibilities.

## 3 Discussion

**FIGURE 4:**
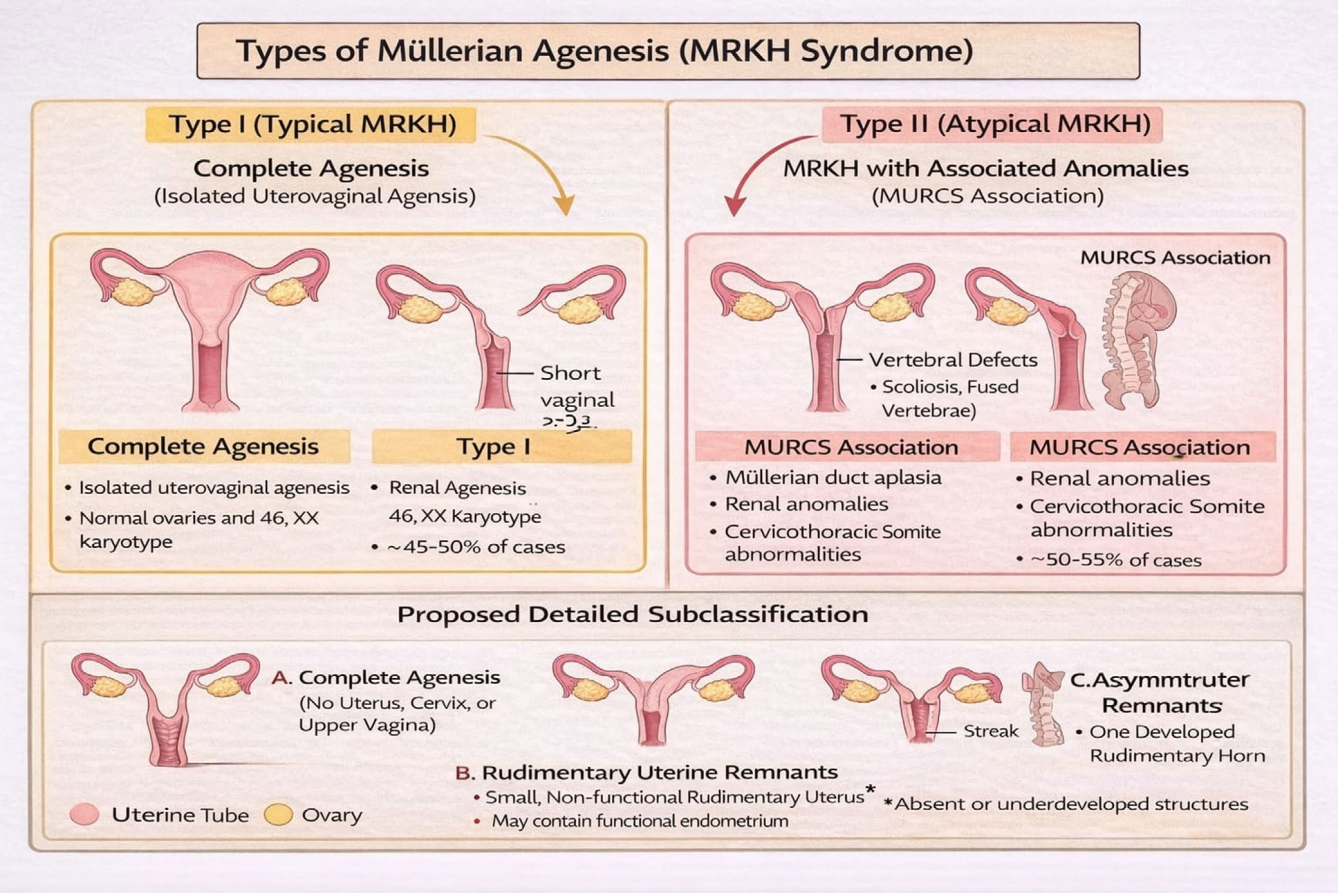
Schematic representation of the types of Müllerian agenesis (MRKH syndrome). Detailed annotated schematic illustrating the spectrum of Müllerian agenesis: Type I (Typical MRKH): Isolated uterovaginal agenesis with absent uterus and upper vagina, normal ovaries, and 46,XX karyotype. Type II (Atypical MRKH/MURCS association): Müllerian agenesis associated with renal anomalies, vertebral or cervicothoracic somite defects, representing ~50%–55% of cases. Proposed detailed subclassification: (A) Complete agenesis with absent uterus, cervix, and upper vagina; (B) Rudimentary uterine remnants, which may or may not contain functional endometrium; (C) Asymmetric remnants with one developed rudimentary horn. Color‐coded annotations highlight key anatomical structures (uterus, ovaries, vagina, fallopian tubes) and differences between types. This figure aids in understanding the anatomical and clinical variations of MRKH syndrome for diagnosis and management.

Müllerian agenesis represents one of the leading causes of primary amenorrhea in adolescents with normal secondary sexual characteristics [1, 7]. The condition results from embryological failure of the Müllerian ducts to develop properly, leading to congenital absence of the uterus and upper vagina while ovarian function remains intact (Figure 4).

Accurate diagnosis requires differentiation from other causes of primary amenorrhea, such as androgen insensitivity syndrome, gonadal dysgenesis, hypothalamic disorders, and obstructive reproductive tract anomalies [3, 12].

**FIGURE 5:**
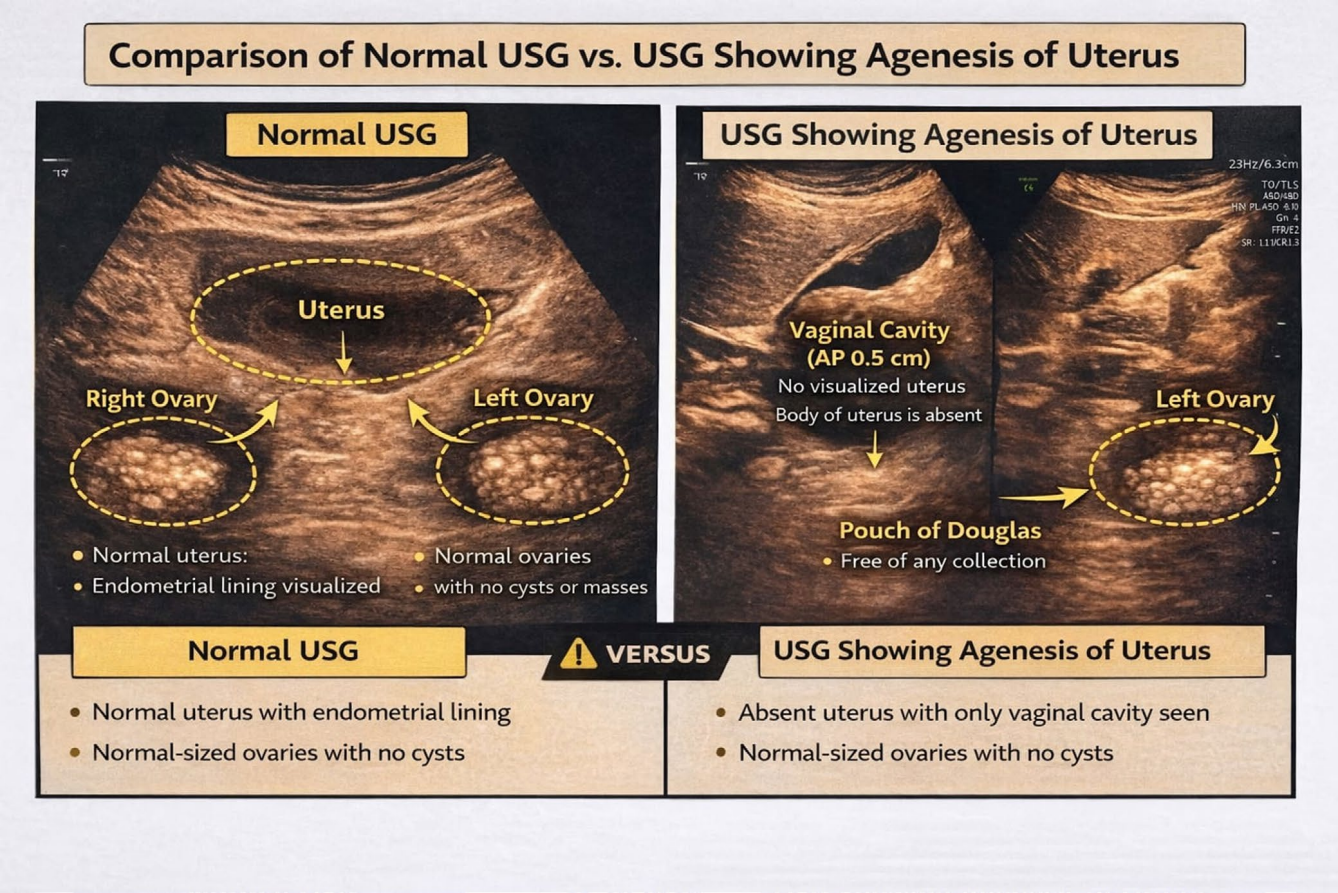
Comparison of normal pelvic ultrasonography and ultrasonography showing Müllerian agenesis. Side‐by‐side ultrasonographic comparison of pelvic anatomy. (a) Normal pelvic ultrasonography demonstrates a well‐defined uterine body with a visible endometrial echo centrally, along with normally positioned bilateral ovaries. (b) Ultrasonography in Müllerian agenesis, showing non‐visualization of the uterine body with identification of a linear structure representing the vaginal cavity (anteroposterior diameter approximately 0.5 cm). Bilateral ovaries are visualized and appear normal in size and echotexture, and the pouch of Douglas is free of any collection.

In high‐income healthcare systems, MRI is frequently used for definitive anatomical evaluation. However, in many resource‐limited settings, ultrasonography combined with careful clinical examination provides sufficient diagnostic information [11] (Figure 5).

**FIGURE 6:**
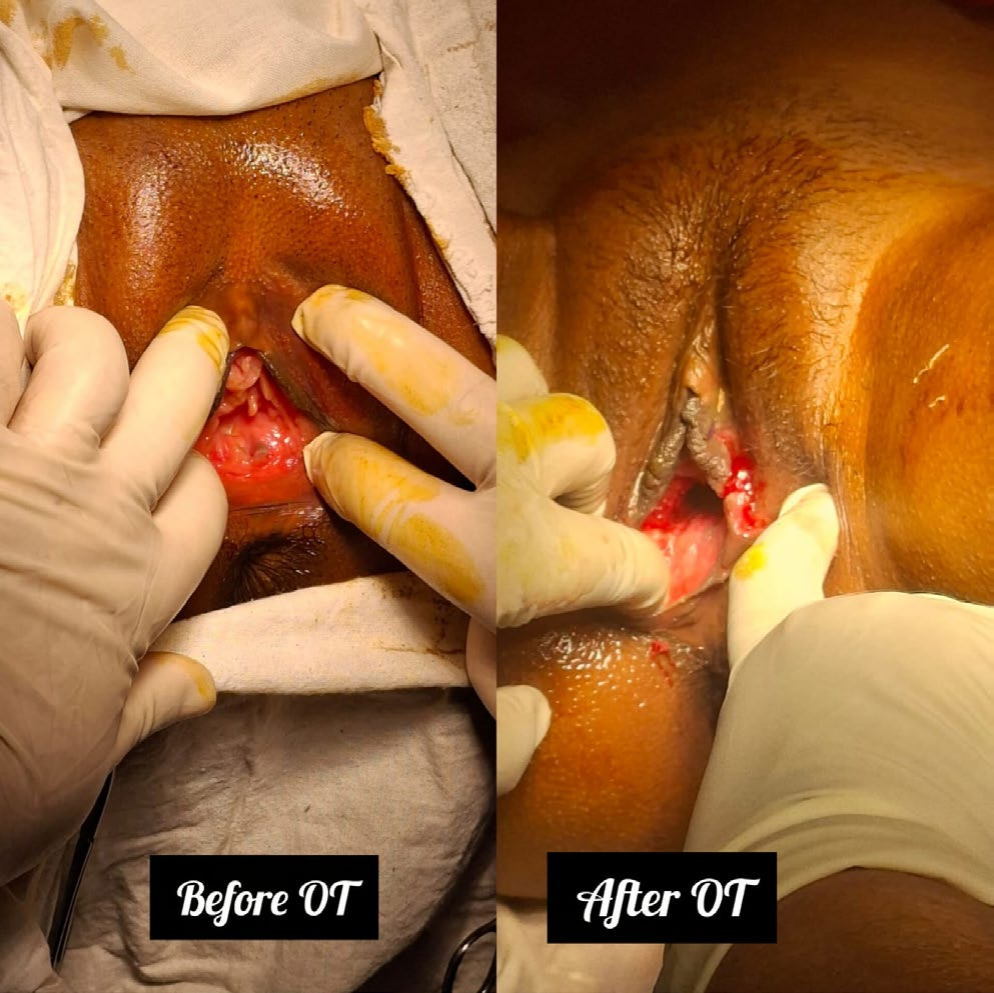
Preoperative and Immediate Postoperative Clinical Appearance Following Vaginoplasty in Müllerian Agenesis. Clinical photographs demonstrate the genital findings before and immediately after surgical vaginoplasty in a patient with Müllerian agenesis (MRKH syndrome). Left panel (Before OT): Preoperative appearance showing a blind vaginal pouch with an absent vaginal canal consistent with Müllerian agenesis. Right panel (After OT): Immediate postoperative appearance following creation of a neovaginal canal. The reconstructed vaginal introitus is visible with fresh surgical margins and layered suturing. Hemostasis is maintained, and the neovaginal cavity has been established. All identifying features are concealed. Images are presented for academic and educational purposes with appropriate patient consent.

Management focuses primarily on the creation of a functional vagina. While non‐surgical vaginal dilation remains the first‐line treatment in many guidelines, surgical vaginoplasty continues to be an effective alternative in settings where conservative approaches are impractical or unsuccessful [4, 5] (Figure 6).

The technique used in the present case demonstrates that successful surgical outcomes can be achieved even in government hospitals with limited resources by using simple and cost‐effective surgical materials.

### 3.1 Psychological Impact and Role of Counseling

The psychosocial impact of MRKH syndrome is significant, particularly during adolescence when individuals are forming their identity and body image [9]. Diagnosis often leads to anxiety regarding femininity, infertility, and future marital relationships.

Research indicates that individuals with MRKH syndrome frequently experience **depression, anxiety, and reduced self‐esteem** following diagnosis [6]. These psychological challenges may be intensified in conservative cultural contexts where menstruation is viewed as an essential marker of womanhood.

Counseling is therefore an essential component of management. Effective counseling should address emotional distress, provide clear information about the condition, and involve family members in the care process. Family‐centered counseling helps reduce stigma and promotes supportive environments for affected individuals.

Advances in reproductive technology, including **in vitro fertilization with gestational surrogacy**, also provide hope for genetic parenthood in the future and may help reduce anxiety related to infertility [5].

### 3.2 Patient Perspective

The patient initially experienced significant anxiety and confusion due to the absence of menstruation while observing that her peers had already attained menarche. She reported feeling different from her friends and avoided discussions related to menstruation or marriage because of embarrassment and fear. Before receiving the diagnosis, she was worried that she might have a serious illness or that she might never be able to marry or lead a normal life.

After detailed counseling by the medical team, the patient and her family gradually understood the nature of the condition. Learning that she was genetically and hormonally a female with functional ovaries provided her considerable emotional relief. She reported feeling reassured after the diagnosis and appreciated the clear explanations provided by the doctors. Following the surgical procedure and postoperative counseling, she expressed satisfaction with the treatment and reported increased confidence and hope regarding her future. The patient and her family expressed gratitude to the healthcare team for their support and care throughout the diagnosis and treatment process.

### 3.3 Significance of This Case

1. Demonstrates successful management of Müllerian agenesis in a **low‐resource government hospital in Bangladesh**.
2. Highlights the Importance of Systematic Evaluation of Primary Amenorrhea.
3. Shows that advanced reconstructive techniques are feasible without high‐end infrastructure.
4. Emphasizes the need for adolescent reproductive health awareness.
5. Encourages strengthening of gynecologic reconstructive services in public sector hospitals.
6. Early, accurate diagnosis prevents unnecessary interventions.
7. Structured counseling reduces psychological trauma and increases self‐independence of the patient.
8. Family‐inclusive communication mitigates stigma.
9. Surgical reconstruction restores anatomical function and confidence.
10. In low‐resource settings, innovative use of locally available materials for vaginal molds demonstrates the feasibility of effective surgical care without expensive infrastructure.
11. Holistic management integrating psychological care is essential for long‐term well‐being.

### 3.4 Limitations

This case report has several limitations. As a single‐patient observation, the findings cannot be generalized to all individuals with MRKH syndrome. Advanced imaging using MRI was not performed due to resource limitations. Additionally, long‐term functional outcomes and psychological adaptation were not assessed because of limited follow‐up duration.

Such constraints are common in clinical reports from resource‐limited healthcare systems [8].

### 3.5 Consent

Written informed consent was obtained from the patient and her legal guardian for publication of this case report and any accompanying clinical images. The patient and her guardian were informed that all personal identifiers would be removed to ensure confidentiality. A copy of the written consent is available for review by the Editor‐in‐Chief of the journal upon request.

## 4 Conclusion

Müllerian agenesis should be suspected in adolescents presenting with primary amenorrhea despite normal development of secondary sexual characteristics. Comprehensive clinical examination, pelvic ultrasonography, and cytogenetic analysis remain essential diagnostic tools [3, 11].

Surgical vaginoplasty can be safely performed in resource‐limited government hospitals when appropriate surgical expertise and postoperative care are available. Equally important is the integration of psychological counseling and family engagement into patient management.

Early diagnosis, culturally sensitive counseling, and multidisciplinary care can significantly improve both physical and psychological outcomes for individuals affected by MRKH syndrome.

### 4.1 CARE Guidelines Statement

This case report was prepared and reported in accordance with the CARE (CAse REport) guidelines to ensure completeness, transparency, and accuracy in the reporting of clinical information Table 1.

**TABLE 1:** Timeline of clinical events.

Time	Event
Age 16	Presentation with primary amenorrhea
Initial visit	Clinical examination and ultrasonography performed
Week 2	Karyotype analysis confirmed 46,XX
Week 3	Diagnosis of MRKH syndrome established
Week 4	Surgical vaginoplasty performed
1 month	Follow‐up showing satisfactory healing
3 months	Continued counseling and good anatomical outcome

## Author Contributions

Iftekhar Ahmed Sakib: 1. Conceptualization 2. Data Curation 3. Formal Analysis 4. Investigation 5. Methodology 6. Project Administration 7. Resources 8. Software 9. Supervision 10. Validation 11. Visualization 12. Writing – Original Draft 13. Writing – Review and Editing. Musarrat Sultana Shumi: 1. Conceptualization 2. Data Curation 3. Formal Analysis 4. Investigation 5. Methodology 6. Project Administration 7. Resources 8. Software 9. Supervision 10. Validation 11. Visualization 12. Writing – Review and Editing. Rozina Sultana: 1. Conceptualization 2. Data Curation 3. Formal Analysis4. Investigation 5. Methodology 6. Project Administration 7. Resources 8. Supervision 9. Validation 10. Visualization 11. Writing – Review and Editing. Ayesa Perveen: 1. Data Curation 2. Formal Analysis 3. Investigation 4. Methodology 5. Project Administration 6. Resources 7. Supervision 8. Validation 9. Visualization 10. Writing – Review and Editing. Nishad Tasnim: 1. Data Curation 2. Formal Analysis 3. Investigation 4. Methodology 5. Project Administration 6. Resources 7. Software 8. Validation 9. Writing – Original Draft. Shirazum Munira: 1. Data Curation 2. Formal Analysis 3. Investigation 4. Methodology 5. Project Administration 6. Resources 7. Software 8. Validation All authors approved the final version of the manuscript and agree to be accountable for all aspects of the work.

## Funding

The authors have nothing to report.

## Data Availability

The data that support the findings of this study are available from the corresponding author upon reasonable request.
